# Effectiveness of Mouthrinse formulated from Aqueous Extract of *Terminalia chebula* on Salivary *Streptococcus mutans* Count and pH among 8- to 12-year-old School Children of Karnataka: A Randomized Clinical Trial

**DOI:** 10.5005/jp-journals-10005-1390

**Published:** 2016-12-05

**Authors:** Madhuchanda Palit, Sundeep K Hegde, Sham S Bhat

**Affiliations:** 1 Reader, Department of Pedodontics, Sardar Patel Postgraduate Institute of Dental and Medical Sciences, Lucknow, Uttar Pradesh, India; 2 Professor, Department of Pedodontics, Yenepoya Dental College Mangaluru, Karnataka, India; 3 Professor and Head, Department of Pedodontics, Yenepoya Dental College Mangaluru, Karnataka, India

**Keywords:** Dental caries, Herbal mouth rinse, *Streptococcus mutans*, *Terminalia chebula.*

## Abstract

**Aim:**

The aim of the present study was to evaluate the anticar-iogenic efficacy of hot and cold aqueous extracts of *Terminalia chebula* against *Streptococcus mutans* as an oral rinse and also to discover the acceptability of the mouthwash in children.

**Settings and design:**

Sixty children between 8 and 12 years with high caries risk were selected.

**Materials and methods:**

10% concentration of hot and cold aqueous extracts were prepared. Children were randomly divided into extract and control group. Baseline salivary samples were taken, and the samples were re-collected at 10, 60, and 90 minutes interval after rinsing. Microbial and pH analysis were done. An acceptability questionnaire was filled.

**Statistical analysis:**

Tukey’s multiple comparison test.

**Results:**

The results show statistically significant difference in S. *mutans* counts at 10, 60, and 90 minutes interval when compared with negative control. However, when the hot and cold extracts were compared, there was no significant difference. Acceptability questionnaire showed 65 to 75% overall acceptability for both types of extract.

**Conclusion:**

Results of this study showed that both types of aqueous extract of *T. chebula* may be used as potential anticariogenic mouthwash with acceptable taste in children.

**How to cite this article:**

Palit MC, Hegde SK, Bhat SS. Effectiveness of Mouthrinse formulated from Aqueous Extract of *Terminalia chebula* on Salivary *Streptococcus mutans* Count and pH among 8- to 12-year-old School Children of Karnataka: A Randomized Clinical Trial. Int J Clin Pediatr Dent 2016;9(4):349-354.

## INTRODUCTION

Dental caries is an irreversible microbial disease of calcified tissues of the teeth, characterized by demineralization of the inorganic portion and destruction of the organic substances of the tooth, which often leads to cavitation. Although there are effective methods known for prevention and management of dental caries, it is a major health problem affecting mankind. It is a complex and dynamic process where a multitude of factors influence and initiate the progression of disease. However, it is uniformly agreed that caries cannot occur without the presence of microorganisms. Evidences implicating its role in etiology of caries is elucidated as germ free animals do not develop caries, antibiotics administered to animals results in reducing the incidence of caries, unerupted teeth do not develop caries, and microorganisms have been demonstrated in enamel and dentinal caries.^1^

*Streptococcus mutans* is considered the primary pathogen in dental caries development.^2^ This cariogenic organism is acquired early in life and the elimination or reduction of such pathogenic bacteria is beneficial in controlling dental caries. Because of this bacterial origin of dental caries, chemotherapeutic agents constitute a reasonable approach toward medical model of management of dental caries.^3^ These agents aid in the removal of microorganisms along with the mechanical plaque control, especially in children who are unable, unwilling, or untrained to practice routine effective mechanotherapy.

Ideal chemotherapeutic plaque control agent should have specificity, substantivity, stability, absence of adverse reactions, and easy to use. No agents have yet been developed that has all these characteristics. In contrary to this, many of the commonly used agents have the adverse effect of alteration of taste, burning sensation, staining, etc., which mandates the search for some newer agents.^4^ Plants or plant products are being used in folk dental practices or prescribed in Unani, homeopathic, or ayurvedic remedies since ages. The beneficial effects of plant products are also scientifically proved. *Terminallia chebula* is also a similar plant-derived medicine with numerous therapeutic effects. It is evident that the ripe fruit of *T. chebula* is valuable in the prevention and treatment of several diseases of the mouth, such as dental caries, spongy and bleeding gums, gingivitis, and stomatitis.^5^ Studies have shown that tannic acid present in *T. chebula* is bacteriostatic or bactericidal to some Gram-positive and Gram-negative pathogens.^6^ Hence, this study aimed to evaluate the antibacterial effect of two different types of aqueous extract of *T. chebula* against *S. mutans* and its acceptability as mouth rinse in children.

## MATERIALS AND METHODS

This study was a single-blinded (microbiologist) randomized control trial conducted in the Department of Pedo-dontics and Preventive Dentistry in collaboration with the Research Center and Department of Microbiology after obtaining Ethical Committee permission. Double blinding could not be done, as it was necessary to inform parents as well as children, in detail regarding the mouthwash and its composition.

### Preparation of the Extract

The dried ripe fruits of *T. chebula* were obtained and ground to fine powder and 10% concentration of hot and cold aqueous extracts were prepared. Cold aqueous extract was prepared by suspending the ripe fruit (400 gm) in 10 times its quantity of sterile distilled water in a round bottomed flask and kept at 4°C for 72 hours. The aqueous extract was then decanted, clarified by filtration through a muslin cloth, and evaporated in a flat-bottomed porcelain dish at 40°C. The dried extract was stored at 4°C prior to use. The extract was suspended in polyethylene glycol (PEG) 400 (20% v/v) and sterile distilled water to give a final concentration of 30% w/v. The concentrated extract was diluted with sterile distilled water to give concentrations of 10% w/v.^7^

The hot aqueous extract was freshly prepared by adding 50 mg of *T. chebula* powder to 50 mL of sterile distilled water and boiling to give a concentration of 10% w/v.^8^

Both hot as well as cold aqueous extract were used as mouth rinse at normal temperature. They differed only in their process of making. Distilled water was used as negative control for both the groups.

### Study Population and Design

All the children coming to the Department of Pedodon-tics for regular checkup were approached. The children between 8 and 12 years were randomly assigned and checked for the inclusion and exclusion criteria. Sixty children were randomly divided into study and control groups. Twenty children were selected as control and 20 each in both the study groups (Flow Chart 1). Information regarding the nature of the study was explained to the parents and children and an informed consent was obtained. Only normal healthy children with full complement of teeth and 1 to 2 established caries lesion were selected. High caries risk assessment was done using AAPD Caries-risk Assessment Tool (CAT).^9^ Children with any systemic diseases, on medication, or any intraoral appliances/prostheses were considered to be the exclusion criteria for the study. The children were randomly divided into study and control group. They were not allowed to eat 2 hours prior to the baseline sample collection. Five milliliters of hot and cold extract were given to the assigned groups respectively 10 minutes after the baseline sample collection and the children were asked to rinse for 1 minute. Salivary sample were collected again at 10, 60, and 90 minutes after rinsing. Unstimulated saliva was allowed to collect at the floor of the mouth and the saliva was collected in two milliliter collection vial.

All children comint to the department of pedodontics for regular checkup were approached

**Flow Chart 1:**
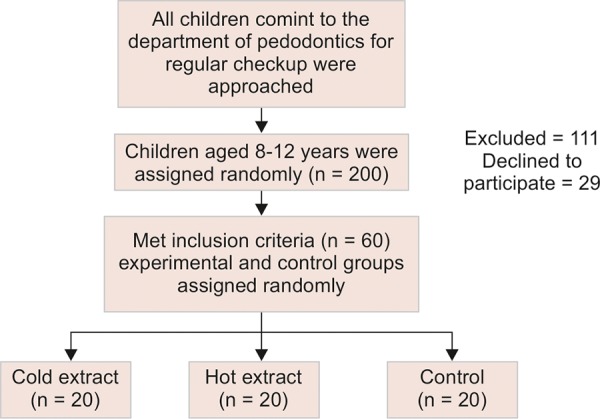
Recruitment and randomization of the participants

pH Analysis

Salivary pH analysis was done by a pH paper in the range of 6 to 8.

### Microbial Analysis

For assessing the microbial content, dilution and spread plate was used.^10^ Salivary microbial analysis was done by diluting each salivary sample 1:10 times.^11^ Each sample was then streaked into Mitis salivarius agar media to determine the total *Streptococcus* count, including the *S. mutans* count. Plates were then incubated for 48 hours at 37°C. The numbers of bacterial colonies were counted in a colony counter to avoid errors.

Acceptability

An acceptability questionnaire was prepared and given to all the children consisting of questions regarding taste, color, odor, and overall acceptance of the extracts. The questionnaire consisted of closed-ended questions regarding whether the extracts were pleasant, acceptable, or unacceptable with respect to color, taste, and odor. Overall acceptability was based on the acceptance of the extracts by the children in terms of color, taste, and odor.

For intragroup comparison of the paired sample, t-test was applied, while for the intergroup comparison between the three groups at different time intervals, Tukey’s multiple comparison tests were applied. All the tests were carried out using Statistical Package for the Social Sciences (SPSS) package in the computer. According to the answers provided by the study subjects, the results of the acceptability questionnaire were expressed in terms of percentage.

**Table 1:** **Table 1:** Intragroup comparison of baseline pH of cold aqueous extract to 10, 30, and 60 minutes (by paired t-test)

				*Mean*		*Std.* *deviation*		*t-value*		*p-value*	
Pair 1		pH before		6.65		0.474		–11.000		<0.001	
		pH 10 min		8.300		0.5375					
Pair 2		pH before		6.65		0.474		–8.135		<0.001	
		pH 60 min		7.90		0.516					
Pair 3		pH before		6.65		0.474		–7.667		<0.001	
		pH 90 min		7.80		0.537					

**Table 2:** **Table 2:** Intragroup comparison of baseline pH of hot aqueous extract to 10, 30, and 60 minutes (by paired t-test)

				*Mean*		*Std.* *deviation*		*t-value*		*p-value*	
Pair 1		pH before		6.50		0.527		–6.228		<0.001	
		pH 10 min		7.750		0.5401					
Pair 2		pH before		6.50		0.527		–6.678		<0.001	
		pH 60 min		7.55		0.158					
Pair 3		pH before		6.50		0.527		–3.791		0.004	
		pH 90 min		7.35		0.337					

**Table 3:** **Table 3:** Intergroup comparison of pH of cold, hot aqueous extracts, and negative control at baseline, 10, 30, and 60 minutes (by Tukey’s multiple comparison test)

*Dependent* *variable*		*(I) group*		*(J) group*		*Mean* *difference* *(I-J)*		*p-value*	
pH before		Cold extract		Hot extract		0.150		0.830	
				Negative control		0.250		0.600	
		Hot extract		Negative control		0.100		0.920	
pH 10 min		Cold extract		Hot extract		0.5500		0.094	
				Negative control		1.9000*		0.000	
		Hot extract		Negative control		1.3500*		0.000	
pH 60 min		Cold extract		Hot extract		0.350		0.283	
				Negative control		1.350*		0.000	
		Hot extract		Negative control		1.000*		0.000	
pH 90 min		Cold extract		Hot extract		0.450		0.174	
				Negative control		1.400*		0.000	
		Hot extract		Negative control		0.950*		0.002	

*Depicts that the mean differences are stastistically significant

**Table 4:** **Table 4:** Intragroup comparison of baseline S. *mutans* colony count of cold aqueous extract to 10, 30, and 60 minutes (by paired t-test)

				*Mean*		*Std.* *deviation*		*t-value*		*p-value*	
Pair 1		*S. mutans* before		717.90		23.101		74.910		<0.001	
		*S. mutans* 10 minutes		167.40		7.933					
Pair 2		*S. mutans* before		717.90		23.101		114.532		<0.001	
		*S. mutans* 60 minutes		195.50		14.057					
Pair 3		*S. mutans* before		717.90		23.101		59.696		<0.001	
		*S. mutans* 90 minutes		269.30		42.859					

**Table 5:** **Table 5:** Intragroup comparison of baseline S. *mutans* colony count of hot aqueous extract to 10, 30, and 60 minutes (by paired t-test)

				*Mean*		*Std.* *deviation*		*t-value*		*p-value*	
Pair 1		*S. mutans* before		674.90		37.534		39.461		<0.001	
		*S. mutans* 10 min		170.80		8.162					
Pair 2		*S. mutans* before		674.90		37.534		44.593		<0.001	
		*S. mutans* 60 min		192.60		22.619					
Pair 3		*S. mutans* before		674.90		37.534		29.271		<0.001	
		*S. mutans* 90 min		233.20		38.169					

## RESULTS

### pH Analysis

When the baseline pH of both the extract groups were compared to the pH at 10, 30, and 90 minutes, there was an increase in pH and it remained alkaline for a period of 90 minutes after rinsing. For cold extract group the mean baseline pH was 6.65 and the mean pH at 90 minutes after the procedure was 7.80 (p < 0.001) (Table 1). For the hot aqueous extract group the mean baseline pH was 6.50 and 7.35 at the interval of 90 minutes after rinsing (p < 0.001) (Table 2). A comparison of pH at 10, 60, and 90 minutes after rinsing between cold extract group and negative control and hot extract group and negative control showed a higher pH of both the extract groups as compared to the negative control (p < 0.001). At 10, 60, and 90 minutes but there was no statistically significant difference between the pH of hot and cold aqueous extract groups (p > 0.05) (Table 3).

### Microbial Analysis

Microbial analysis of salivary samples indicated that there was reduction in salivary *S. mutans* colony at 10, 60, and 90 minutes after rinsing in both the extract groups compared with the baseline *S. mutans* colony count (p < 0.001) (Tables 4 and 5). When the hot and cold aqueous extract groups were compared with the negative control, there was a lower *S. mutans* colony count at 10, 60, and 90 minutes (p < 0.001). However, when both the extract groups were compared at 10, 60, and 90 minutes, there was no difference in *S. mutans* colony count (p > 0.05) (Table 6).

**Table 6:** **Table 6:** Intergroup comparison of S. *mutans* colony count of cold, hot aqueous extracts, and negative control at baseline, 10, 30, and 60 minutes (by Tukey’s multiple comparison test)

*Dependent* *variable*		*(I) group*		*(J) group*		*Mean* *difference* *(I-J)*		*p-value*	
*S. mutans*		Cold extract		Hot extract		43.000		0.026	
before				Negative control		13.400		0.666	
		Hot extract		Negative control		–29.600		0.154	
*S. mutans*		Cold extract		Hot extract		–3.400		0.941	
10 min				Negative control		–544.900		0.000	
		Hot extract		Negative control		–541.500		0.000	
*S. mutans*		Cold extract		Hot extract		2.900		0.961	
60 min				Negative control		–541.500		0.000	
		Hot extract		Negative control		–544.400		0.000	
*S. mutans*		Cold extract		Hot extract		36.100		0.136	
90 min				Negative control		–454.700		0.000	
		Hot extract		Negative control		–490.800		0.000	

**Table 7:** **Table 7:** Results of acceptability questionnaire

		*Number of percentage of subjects* * finding the extract acceptable*			
*Acceptability*		*Cold aqueous* *extract (n = 20)*		*Hot aqueous* *extract (n = 20)*	
Color		18 (90%)		19 (95%)	
Odor		17 (85%)		19 (95%)	
Taste		11 (55%)		13 (65%)	
Overall acceptability		13 (65%)		15 (75%)	

### Acceptability

The results of acceptability questionnaire showed that out of the three categories of pleasant, acceptable, and unacceptable, almost all the subjects found the extracts acceptable with respect to color, odor, taste, and overall acceptability. None of the children found taste and overall acceptability of both the extract group pleasant. Out of the 20 subjects in each cold and hot extract group, color was acceptable to 18 (16 acceptable, 2 pleasant) and 19 (16 acceptable, 3 pleasant) subjects, odor was acceptable to 17 (15 acceptable, 2 pleasant) and 19 (17 acceptable, 2 pleasant) subjects, and taste was acceptable to 11 and 13 subjects respectively. Overall acceptability was 65 and 75% respectively for cold and hot extracts (Table 7).

## DISCUSSION

For thousands of years, medicinal plants have played an important role throughout the world in treating and preventing various diseases. *T. chebula,* also known as Haritaki, chebulic Myrobalan, or inknut, is a similar plant-derived medicine, which has been used since antiquity to control numerous human diseases. It is always listed first in the Ayurvedic Materia Medica in India. It exerts a wide range of pharmacological effects, including antibacterial, antiviral, antifungal, antioxidant, antiarthritic, antiaging properties.^5,6,12-15^ It also exhibits good hepatoprotection and nephroprotection against acetaminophen toxicity.^16^ Literature review reveals abundant evidence for the use of plants and plant products in preventing dental caries.^17,18^ In our study, we have selected *T. chebula* - to evaluate the efficacy of its extract as an anticariogenic mouth rinse in children.

Tannins are a group of polymeric phenolic compounds and they are the major constituent of *T. chebula* responsible for its antimicrobial property against *S. mutans.* Tannins inhibit enzyme glucosyltransferase by virtue of some tannin-protein interaction, hence affecting dextran-induced aggregation of *S. mutans* on the tooth surface. It also inhibits the acid production and sucrose-mediated adherence of *S. mutans.^5^* In the present study, we have used an aqueous solution of *T. chebula* as most of the plant products are soluble in it. Methanol or ethanol extract could have given a better result, but the adverse effect of alcoholic extracts in children has further warranted us to _19_ use aqueous extracts.^19^

We used both hot as well as cold aqueous extracts at 10% concentration according to the works of Jagtap and Karkera.^5^ They suggested that at concentrations of 10%, the extract had an immediate effect on the salivary bacteria, and this effect was retained for 3 hours. However, at a lower concentration it could not inhibit *S. mutans* growth. Hence the rinsing of the mouthwash with water should be avoided, as it will cause dilution of the mouthwash and might decrease its efficiency by reducing the concentration.

The hot and cold aqueous extract groups differed in their preparation method. Hot extract was prepared according to the works of Nayak et al,^8^ while the methodology of Carounanidy et al^7^ was followed to prepare cold aqueous extract. Both the extracts, however, were used as a mouth rinse at normal temperature.

In hot aqueous extract group, the extract were freshly prepared, hence solvent was not needed; whereas in the cold aqueous extract group, PEG was used as solvent. Results of our study showed that both types of extracts at similar concentrations were equally effective in reducing *S. mutans* count. Hence, in the absence of pharmaceutical grade PEG, hot aqueous extract can also be used with equal effectiveness, however, the preparation of fresh extract every time will be cumbersome for larger study population.

Unstimulated salivary pH was analyzed using color indicator pH strips. Unstimulated saliva was used, as resting salivary pH plays a major role in caries initiation. Our study showed an increase in pH up to 90 minutes after rinsing. Nayak et al^8^ and Carounanidy et al^7^ noted an increase in pH up to 1 hour and 45 minutes. Jagtap and Karkera^5^ noted an increase at 2, 3, and 5 hours after rinsing. Hence, both the extracts have a high substanti-vity, which is essential for an effective mouthwash.

The present study showed a statistically significant reduction in *S. mutans* colony count at 10 minutes after rinsing with both types of aqueous extracts and this decrease in the colony remained below the baseline even after 90 minutes. Similar sustained decrease in salivary bacteria for a period of 3 hours was noted by Jagtap and Karkera.^5^ According to Nayak et al,^8^ there was a observed reduction in the colony count up to 1 hour after rinsing. This reduction is mainly because tannic acid binds strongly to the carboxyl groups of the salivary glyco-proteins present on the pellicle and hence prolonging its antimicrobial activity against *S. mutans.^6^*

For counting the *S. mutans* colony, we have used 1:10 dilution,^10,11^ whereas Nayak et al^8^ have used 1:100 dilutions. Decreasing the dilution (1:10) as done in our study will give a more crowded colony as compared with higher dilution (1:100),^10^ which might be the reason for the difference in mean between the two studies. Since the crowded colony would be difficult to count, we have used a micro-bial colony counter, which has as well reduced the error.

**Graph 1:**
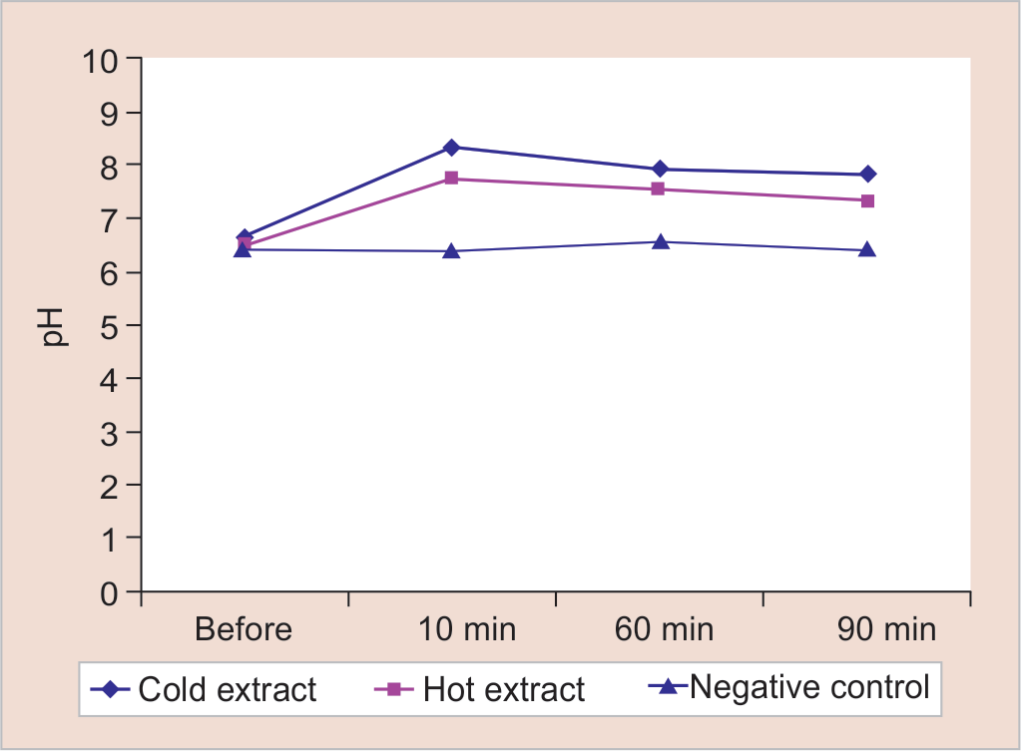
Change in pH up to 90 minutes after rinsing

**Graph 2:**
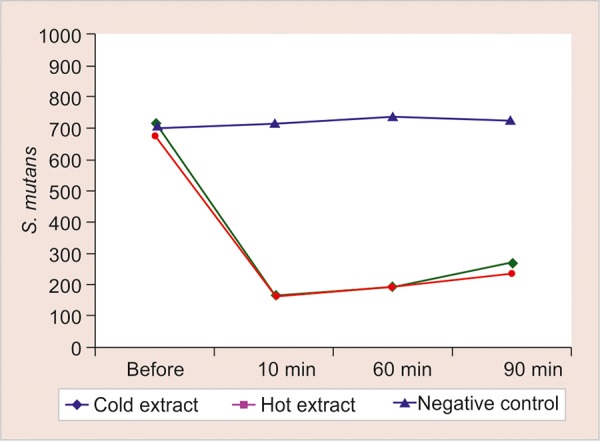
Change in *S. mutans* colony count up to 90 minutes after rinsing

Salivary *S. mutans* colony count and pH were evaluated at different time intervals to evaluate its effect for a prolonged time. The result of our study showed that there was definite decrease in all the parameters even after 90 minutes as compared with baseline, but there was a gradual increase at 10, 60, and 90 minutes (Graphs 1 and 2). Hence, further studies for a longer duration might help us to determine the dose interval of the mouth rinse, i.e., how frequently it should be prescribed to obtain a sustained reduced oral *S. mutans* count.

To measure the acceptability of both the hot and cold aqueous extracts, we have given closed-ended questionnaire to the children. The extracts were categorized as “pleasant,” “acceptable,” or “unacceptable” in terms of “color,” “odor,” “taste,” and “overall acceptability.” In our study, result of the acceptability questionnaire indicated that the color, odor, and taste was acceptable at 90, 85, and 55% for cold extract, whereas for hot extract it was 95, 95, and 65% respectively. Majority of the children found taste, color, and odor as “acceptable.” The overall acceptability was also “acceptable” to most (65% for cold and 75% for hot extract) of the children. As only 5 children out of 40 for both the extract groups found overall acceptability as “pleasant” and none of them found it “unacceptable,” the overall acceptability for the categories “pleasant” and “unacceptable” was not considered. These results were similar to the results by Nayak et al.^8^ Hence, the overall acceptability results indicated that the extracts may be acceptable to most children.

Ours is the first study where *T. chebula* mouth rinse has been used in children. We have studied the effects of both types of *T. chebula* aqueous extract; a comparison study of the rinse over conventionally available mouth rinses was not done. Hence, further studies are required to compare the aqueous extracts to the conventionally available mouth rinses in children.

The present study is limited to a short duration of 90 minutes. Further research should also focus on long-term use of the mouth rinses on larger sample size as well as dose determination.

*T. chebula* may therefore be used as an anticariogenic mouthwash in children due to its effectiveness in reducing oral load of *S. mutans.* It is economical, easy to prepare, has good substantivity, and also provides multiple beneficial health effects. It may also be well accepted by the children, however long-term study with larger sample size and dose determination is further required.

### Importance to Pediatric Dentistry

- *T. chebula* may be used as an effective oral rinse in children with high caries risk to reduce the oral load of *S. mutans.*
